# Polystyrene Chain Growth from Di-End-Functional Polyolefins for Polystyrene-Polyolefin-Polystyrene Block Copolymers

**DOI:** 10.3390/polym9100481

**Published:** 2017-10-02

**Authors:** Chung Sol Kim, Seung Soo Park, Sung Dong Kim, Su Jin Kwon, Jun Won Baek, Bun Yeoul Lee

**Affiliations:** Department of Molecular Science and Technology, Ajou University, Suwon 443-749, Korea; fubu50f@ajou.ac.kr (C.S.K.); zerg113@ajou.ac.kr (S.S.P.); ksd92v@hanmail.net (S.D.K.); ksj9355@ajou.ac.kr (S.J.K.); btw91@ajou.ac.kr (J.W.B.)

**Keywords:** coordinative chain transfer polymerization, di-end-functional polyolefin, block copolymer, thermoplastic elastomer

## Abstract

Triblock copolymers of polystyrene (PS) and a polyolefin (PO), e.g., PS-*block*-poly(ethylene-*co*-1-butene)-*block*-PS (SEBS), are attractive materials for use as thermoplastic elastomers and are produced commercially by a two-step process that involves the costly hydrogenation of PS-*block*-polybutadiene-*block*-PS. We herein report a one-pot strategy for attaching PS chains to both ends of PO chains to construct PS-*block*-PO-*block*-PS directly from olefin and styrene monomers. Dialkylzinc compound containing styrene moieties ((CH_2_=CHC_6_H_4_CH_2_CH_2_)_2_Zn) was prepared, from which poly(ethylene-*co*-propylene) chains were grown via “coordinative chain transfer polymerization” using the pyridylaminohafnium catalyst to afford di-end functional PO chains functionalized with styrene and Zn moieties. Subsequently, PS chains were attached at both ends of the PO chains by introduction of styrene monomers in addition to the anionic initiator Me_3_SiCH_2_Li·(pmdeta) (pmdeta = pentamethyldiethylenetriamine). We found that the fraction of the extracted PS homopolymer was low (~20%) and that molecular weights were evidently increased after the styrene polymerization (Δ*M*_n_ = 27–54 kDa). Transmission electron microscopy showed spherical and wormlike PS domains measuring several tens of nm segregated within the PO matrix. Optimal tensile properties were observed for the sample containing a propylene mole fraction of 0.25 and a styrene content of 33%. Finally, in the cyclic tensile test, the prepared copolymers exhibited thermoplastic elastomeric properties with no breakage up over 10 cycles, which is comparable to the behavior of commercial-grade SEBS.

## 1. Introduction

Polyolefins (POs), including polyethylene (PE) and polypropylene (PP), are the most abundantly produced polymers worldwide, with annual production of ~120 million metric tons. The subtle architecture of the PO chains is of particular interest in the bulk polyolefin industry. For example, sequence control to form olefin block copolymers (OBCs) is a formidable task in olefin polymerizations [1,2,3,4,5]. Following the commercialization of OBCs by the Dow Chemical Company [6,7,8], Coates et al*.* recently reported the preparation of PE/iPP (iPP = isotactic polypropylene) multiblock copolymers to enhance capabilities of plastic recycling [1]. In addition, connection of PO chains with other polymers such as polystyrene (PS) or polar monomer-based polymers to prepare diverse PO-based block copolymers has also been reported [9,10,11,12,13,14,15,16,17], with a typical example being PO-PS block copolymers, e.g., PS-*block*-poly(ethylene-*co*-1-butene)-*block*-PS (SEBS), which are commercially produced on a scale of several hundred thousand metric tons per year. Due to its thermoplastic elastomer properties, SEBS is commonly employed in commodities such as rubbers and plastics [18,19,20,21,22], and its use in new specialized areas is also receiving growing interest [23,24,25,26,27,28,29]. In industry, SEBS is not produced directly from olefin and styrene monomers, but instead by a two-step process that involves the costly hydrogenation of the PS-*block*-polybutadiene-*block*-PS (SBS) copolymer produced by the living anionic polymerization of styrene and butadiene [30,31]. Indeed, the hydrogenation of polymers is not an easy process, so this requirement adds significantly to the manufacturing cost of SEBS.

**Scheme 1:**
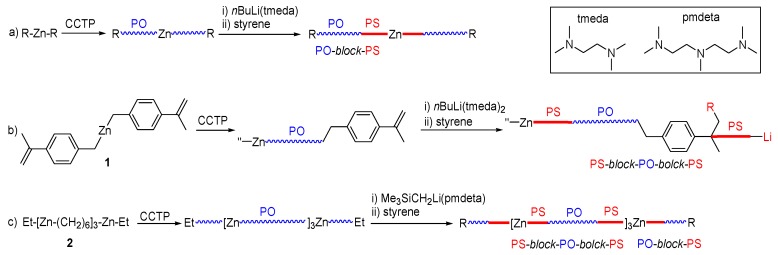
(**a**–**c**) Previously attempted strategies for preparation of polyolefin-polystyrene (PO-PS) block copolymers.

**Scheme 2:**
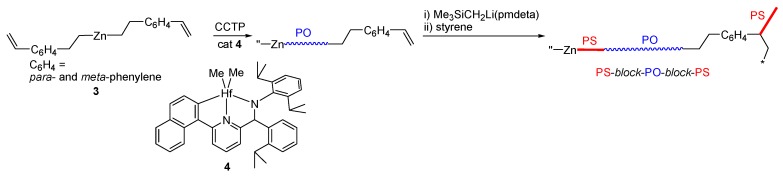
Strategy employed herein for the preparation of PS-*block*-PO-*block*-PS.

In this context, we previously designed a strategy for the direct synthesis of PO-PS diblock copolymers from olefin and styrene monomers that involved coordinative chain transfer polymerization (CCTP) and subsequent anionic polymerization of styrene in a one-pot system (Scheme 1a) [32]. CCTP is an established technology that is actively employed in olefin polymerization reactions where precise architectural design and end group functionalization are required [33,34,35,36,37,38,39,40]. In the CCTP process, PO chains are grown from a dialkylzinc (e.g., Et_2_Zn) to form (polymeryl)_2_Zn species. We discovered that PS chains can be subsequently grown from the generated (polymeryl)_2_Zn by feeding styrene monomer in addition to an anionic initiator such as *n*BuLi·(tmeda) (tmeda = tetramethylethylenediamine) or, preferably, Me_3_SiCH_2_Li·(pmdeta) (pmdeta = pentamethyldiethylenetriamine). We then attempted the preparation of SEBS-like PS-*block*-PO-*block*-PS triblock copolymers using a dialkylzinc species (i.e., [4-(isopropenyl)benzyl]_2_Zn, **1**) bearing the α-methylstyrene moiety, from which PO chains were grown by CCTP to generate di-end functional POs functionalized with Zn and α-methylstyrene moieties (Scheme 1b) [41]. PS chain growth from both ends was attempted, but anionic polymerization at the α-methylstyrene moiety was sluggish in the presence of the dialkylzinc species. We then examined the preparation of a multinuclear zinc species, i.e., Et-[Zn-(CH_2_)_6_]_3_-Zn-Et, from which the PO chain and subsequently the PS chain were biaxially grown in a one-pot process to generate the desired PS-*block*-PO-*block*-PS chains (Scheme 1c) [42]. However, the generation of PS-*block*-PO diblock chains (25 wt %) from the Zn-Et moiety in Et-[Zn-(CH_2_)_6_]_3_-Zn-Et was inevitable, and the molecular weight was reduced by one fifth after quenching, thereby leading to viscosity problems during the production of high molecular weight polymers. Thus, we herein report an alternative method for the construction of PS-*block*-PO-*block*-PS copolymers (Scheme 2), which is expected to provide a number of advantages over previously reported methods.

## 2. Experimental Section

All manipulations were performed under an inert atmosphere using a standard glove box and Schlenk techniques. Methylcyclohexane was purchased from Sigma-Aldrich (St. Louis, MO, USA) and purified over a Na/K alloy. The ethylene/propylene gas mixture was purified over trioctylaluminum (0.6 M in methylcyclohexane) in a bomb reactor (2.0 L). Styrene purchased from Sigma-Aldrich was purified by stirring over *n*BuLi·AlEt_3_ (2.3 mmol/300 g-styrene) for 3 days. Pmdeta was purchased from Sigma-Aldrich and purified over CaH_2_. The ^1^H NMR (400 MHz) spectra were recorded using a Varian Mercury Plus 400 instrument. The gel permeation chromatograms (GPC) of the PS homopolymers were obtained in toluene at 40 °C using a Waters Millennium apparatus, while those of the POs and block copolymers were obtained in 1,2,4-trichlorobenzene at 160 °C using a PL-GPC 220 system equipped with a RI detector and two columns (PLgel mixed-B 7.5 × 300 mm from Varian (Polymer Lab, Palo Alto, CA, USA)). Transmission electron microscope (TEM) images were recorded on a Carl Zeiss LIBRA 120 instrument. Complex **4** was prepared according to a previously reported literature method [43].

### 2.1. Preparation of (CH_2_=CHC_6_H_4_CH_2_CH_2_)_2_Zn *(**3**)*

**Figure 1:**
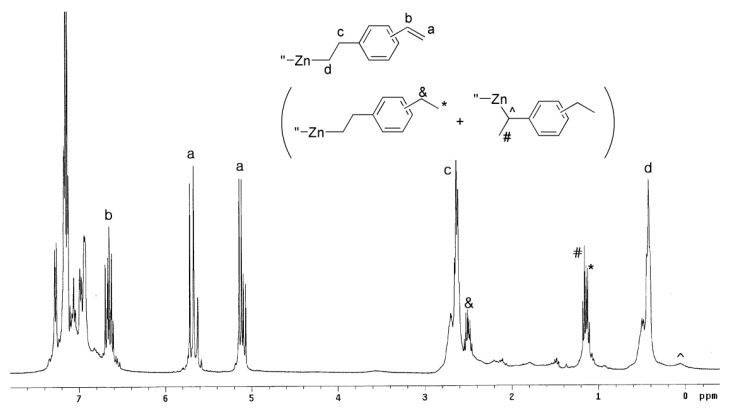
^1^H NMR spectrum of **3** in C_6_D_6_.

A solution of borane dimethyl sulfide (0.24 g, 3.2 mmol) was added dropwise to Et_3_B (0.94 g, 9.6 mmol) at room temperature with stirring. After stirring for 90 min, the resulting solution was added dropwise to a solution of divinylbenzne (80% technical grade, 3.8 g, 28.8 mmol) in anhydrous diethyl ether (5.3 mL) at −40 °C. After stirring for 1 h at room temperature, the volatile solvents were removed using a vacuum line at room temperature, and Et_2_Zn (0.83 g, 6.7 mmol) was added. The reaction vessel was then connected to a distillation apparatus and the system was thoroughly evacuated at −20 °C. Under these vacuum conditions at 0 °C, the transiently generated Et_3_B was selectively transferred for 5 h from the vessel to a receiving flask that was cooled using a dry ice/acetone bath. Excess Et_2_Zn and divinylbenzene were then removed from the reaction pot to the receiving flask by vacuum distillation at 40 °C, after which the reaction system was transferred to a glove box and methylcyclohexane (10 g) was added. The resulting insoluble black material was removed by filtration using Celite, and the obtained filtrate containing the desired **3** employed in the CCTP without further purification. The concentration of this solution was determined to be 72.3 mg/g by measuring the weight of solute following the removal of solvent from a portion of the solution (0.500 g), which gave an overall yield of 0.87 g **3** (83%). As indicated in Figure 1, divinylbenzene employed in this step was a mixture of *para*- and *meta*-isomers and also contained traces of ethylvinylbenzene, thereby resulting in a complicated ^1^H NMR spectrum for the product. It should be also noted that Et_2_Zn is volatile and pyrophoric, and thus any collected solvent and byproduct were carefully quenched with water following dilution in mineral oil.

### 2.2. Typical Procedure for the Synthesis of Block Copolymers (Entry 5 in Table 1)

To remove any catalyst poisons from the reactor vessel, a bomb reactor (125 mL) was charged with a solution of Me_3_Al (14.4 mg, 200 μmol-Al) in methylcyclohexane (17.0 g), stirred for 1 h at 100 °C using a heating mantle, and the solution removed from the reactor vessel using a cannula. After completing this washing step, the reactor was charged with a solution of (CH_2_=CHC_6_H_4_CH_2_CH_2_)_2_Zn (**3**, 45.0 mg, 150 μmol) in methylcyclohexane (45.0 g) under an inert atmosphere, and the temperature was set to 70 °C. The catalyst stock solution was prepared by reacting Hf complex **4** (18.1 mg, 25.0 μmol) with [(C_18_H_37_)_2_MeNH]^+^[B(C_6_F_5_)_4_]^−^ (30.4 mg, 25.0 μmol) in benzene (4.0 g). This catalyst stock solution (648 mg, 4.0 μmol-Hf complex) was subsequently injected into the reactor vessel using a syringe, and the system was immediately charged with a gaseous mixture of ethylene/propylene at 20 bar pressure, which resulted in a temperature increase to ~125 °C within 5 min due to the exothermic reaction, despite cooling of the reactor with a fan. Following this initial increase, the reaction temperature began to decrease gradually due to catalyst deactivation, and was maintained between 95 °C and 100 °C. The pressure within the system gradually decreased, finally reaching 16 bar, due to consumption of the monomers, while the stirring rate decreased gradually from 300 to 130 rpm due to the formation of a thick viscous solution. After allowing the polymerization process to continue for 45 min, the remaining gas was vented off. During this procedure, the polymer solution swelled up to plug the valve and was withdrawn for GPC and ^1^H NMR analyses. When the temperature has reduced to 90 °C, a solution of Me_3_SiCH_2_Li·(pmdeta), prepared by mixing Me_3_SiCH_2_Li (14.1 mg, 0.150 mmol) and pmdeta (26 mg, 0.150 mmol) in methylcyclohexane (1.0 g), was added. The temperature was then maintained at 90 °C for 30 min with stirring, after which time styrene (7.8 g, 750 mmol) was injected. The temperature of the system was controlled in the range of 100–110 °C using a heating mantle, and the viscosity of the reaction mixture gradually increased over 5 h to the point where stirring was impossible. The complete conversion of styrene was confirmed by ^1^H NMR analysis of an aliquot of the obtained mixture, after which acetic acid and ethanol were successively injected. The obtained polymer mass was then dried overnight in a vacuum oven at 180 °C to give a mass of 24.1 g. After dissolving the polymer (2.8 g) in boiling chloroform (30 g), acetone (60 g) was added to precipitate the block copolymers. The PS-homopolymers, which were freely soluble in the chloroform/acetone cosolvent, were isolated initially via filtration. The resulting solution was then subjected to rotary evaporation and the residual solvent was completely removed in a vacuum oven at 80 °C over the course of several hours to obtain the PS homopolymers (0.21 g). The samples containing the PS homopolymers were then analyzed by GPC, TEM, DSC, and tensile tests.

### 2.3. High-Temperature GPC Studies

Sample solutions (200 μL) containing 3000 ppm of the polymer substances were eluted in 1,2,4-trichlorobenzene at a flow rate of 1.0 mL/min at 160 °C, and the mobile phase was stabilized with 2,6-di-*tert*-butyl-4-methylphenol (0.04%). The PS-based molecular weight distributions were calculated from a calibration curve on the basis of narrow PS standards (*M*_p_: 480; 915; 4715; 10,009; 20,681; 73,143; 197,736; 419,672; 1,047,000; 2,906,000; 4,714,000 Da). For calculation of the PO-based molecular weight distributions, the PS standard molecular weights (*M*_PS_) were converted to PE equivalents (*M*_PE_) using the reported Mark-Houwink-Sakurada parameters for PS (*K* = 0.000121; *a* = 0.707) and PE (*K* = 0.000406; *a* = 0.725) according to the equation: *M*_PE_ = [(0.000121/0.000406) × *M*_PS_^(1+0.707)^]^(1/(0.725+1))^ = 0.495 × *M*_PS_^0.990^, and further to PO equivalents using the equation *M*_PO_ = *M*_PE_/(1 − *S*) where S is the mass fraction of the CH_3_-side chains (i.e., *S* = (15 × *F*_C3_)/[(1 − *F*_C3_ ) × 28 + (*F*_C3_ × 42)]) [7,44].

### 2.4. Sample Preparation for Transmission Electron Microscopy (TEM)

The triblock copolymer (5 mg) was dissolved in toluene (5 mL) at 100 °C, and a drop of the hot solution was loaded onto a carbon-coated copper TEM grid (200 mesh). Following slow evaporation of the solvent at room temperature overnight, the sample was annealed in an oven over 150 °C for 6 h, prior to staining with RuO_4_ by suspending the coated TEM grid for 30 min in a closed chamber containing an aqueous solution of RuO_4_, which was prepared by reacting RuO_2_ (30 mg) with NaIO_4_ (0.20 g) in water (5 mL) at 0 °C for 4 h.

### 2.5. Tensile Tests

The obtained polymer samples were compressed between hot plates at 135 °C and 5 MPa for 20 min followed by similar treatment at 10 MPa for 100 min. The obtained polymer films (~1 mm thickness) were cut into pieces (100 × 10 mm^2^), and tensile tests were performed according to the ASTM D882 standard test method using a UTM (WL2100, KOPTRI, Seoul, Korea) at a drawing rate of 500 mm/min with a gauge length of 50 mm at 25 (±2) °C and 45 (±5)% humidity. In the cyclic tensile test, each specimen was extended over 10 cycles to half of the distance of the breakage length measured in the tensile test.

## 3. Results and Discussion

### 3.1. Strategy for the Preparation of PS-Block-PO-Block-PS

The strategy employed herein for preparation of the PS-*block*-PO-*block*-PS is similar to that attempted using the dialkylzinc species bearing the α-methylstyrene moiety (Scheme 1b versus Scheme 2). In the previous attempt (Scheme 1b), a typical *ansa*-metallocene *rac*-[Me_2_Si(2-methylindenyl)_2_]ZrCl_2_ was used as the catalyst in the CCTP process. In that case, the styrene moieties took part in the CCTP either as chain transfer agents or as comonomers [9,45], and so a dialkylzinc compound bearing the less reactive α-methylstyrene moiety was required. Although the α-methylstyrene moiety remained intact during the CCTP performed using *rac*-[Me_2_Si(2-methylindenyl)_2_]ZrCl_2_, it was sluggishly involved in the subsequent anionic styrene polymerization. Thus, we herein attempted the use of a dialkylzinc species bearing a styrene moiety instead of an α-methylstyrene moiety (i.e., **1** versus **3**) in addition to the use of pyridylaminohafnium catalyst **4** instead of *rac*-[Me_2_Si(2-methylindenyl)_2_]ZrCl_2_, as the former is reportedly a more efficient catalyst for the CCTP and prevents the undesirable β-elimination process [1,7,8,42,46,47]. It should also be noted that the styrene moiety did not interfere in the CCTP performed using **4**. Indeed, the presence (or absence) of the styrene monomer during the ethylene/propylene copolymerization reaction using **4** had no effect on the transformation; yields were identical (14.5 and 14.2 g in the presence and in the absence of styrene, respectively) and the GPC data were either identical (*M*_n_ = 29800, *M*_w_ = 41,800 in the presence of styrene; *M*_n_ = 29,500, *M*_w_ = 41,100 in the absence of styrene). Moreover, no styrene-related signals were observed in the ^1^H NMR spectrum of the PO generated in the presence of styrene (Figure S1). In the other way, the styrene signals were observed at 7.2–5.0 ppm region (Figure S2) in the ^1^H NMR spectrum of a low molecular weight poly(ethylene-*co*-propylene), which was synthesized by feeding a high amount of **3** (Zn = 500 μmol) and by cutting the polymerization at an early stage (5 min).

**Scheme 3:**
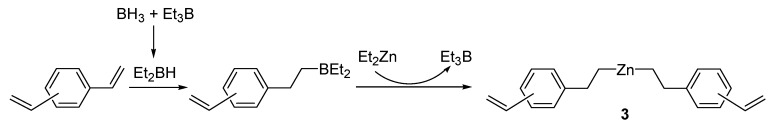
Synthesis of the dialkylzinc species containing styrene moieties.

Preparation of the dialkylzinc species containing styrene moieties (**3**) was carried out through alkyl exchange between Et_2_Zn and the hydroboration product of divinylbenzene (Scheme 3) [42,48]. During the dynamic alkyl exchange process, the transient Et_3_B species was removed by evacuation at 0 °C, eventually leading to formation of the desired product, 3. As the employed commercial grade divinylbenzene is a mixture of para- and meta-isomers also containing traces of ethylvinylbenzene (80% purity), the obtained ^1^H and ^13^C NMR spectra were not clean; however, the major signals could be clearly assigned to compound **3** (Figure 1 and Figure S3). In addition, it should be noted that **1** was synthesized by metathesis between ZnCl_2_ and the corresponding Grignard reagent, in which contamination by a small quantity of a magnesium-containing compound led to catalyst poisoning, so tedious recrystallization was required prior to use in the CCTP [49]. In contrast, **3** prepared from alkyl exchange between Et_2_Zn and borane was suitable for use in the CCTP without any additional purification.

### 3.2. Synthesis of PS-Block-PO-Block-PS

Following the development of a suitable strategy for the synthesis of PS-*block*-PO-*block*-PS, the CCTP reaction was performed in the presence of **3** (150 μmol) using **4** (4.0 μmol) activated with [(C_18_H_37_)_2_MeNH]^+^[B(C_6_F_5_)_4_]^−^ (1.0 equiv) as the catalyst. During this process, a ethylene/propylene gas mixture was fed into the system, and the polymerization temperature was maintained at 95–110 °C to prevent precipitation of the generated polymers. In addition, the propylene mole fraction (i.e., *F*_C3_ = [C3]/([C2] + [C3])) was varied between 0.22 and 0.30 for the POs by controlling the propylene/ethylene ratio in the feed gas (Table 1). Indeed, the measured molecular weights (*M*_n_, ~50 kDa) of the POs generated in CCTP process, after conversion to PO-equivalents by universal calibration, corresponded with the expected values calculated using the formula: PO weight (g)/(2 × Zn (mol)), and the molecular weight distribution was narrow (i.e., *M*_w_/*M*_n_ = 1.9) (Table 1). These results indicated that the CCTP proceeded successfully with the generation of di-end functional PO chains from **3**, where the generation of mono-end functional and non-functional PO chains was suppressed. When CCTP reaction was performed under the identical conditions using Et_2_Zn instead of **3**, the measured molecular weight (*M*_n_, 34 kDa, *M*_w_/*M*_n_, 1.69) of the generated PO deviated from the expected value calculated using the formula (i.e., 15.4 g/(2 × 150 μmol) = 51 kDa). The measured value corresponded with the value calculated based upon the assumption that PO chains were grown from the alkyl sites on both Zn and Al species (i.e., 15.4 g/(2 × 150 μmol + 3 × 50 μmol) = 34 kDa). We guess that the rapid alkyl exchange occurred between trioctylaluminum and diethylzinc, generating ethyl(dioctyl)aluminum, diethyl(octyl)aluminum, and triethylaluminum species, which participated in the CCTP process because of the loss of steric congest on the Al center. Such alkyl exchange might not occur between **3** and trioctylaluminum. Trioctylaluminum did not participate in the CCTP process, just acting as a scavenger.

**Scheme 4:**
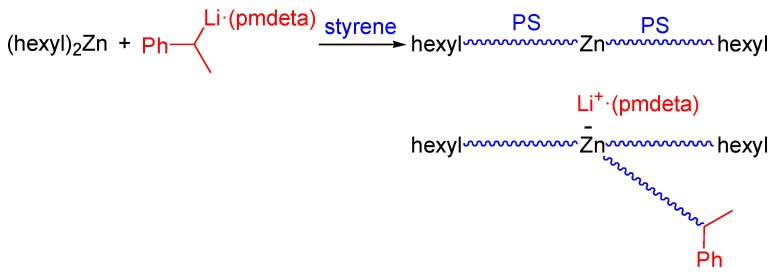
PS chain growth from (hexyl)_2_Zn by the action of styryl-Li·(pmdeta).

In previous studies, Me_3_SiCH_2_Li·(pmdeta) was introduced as an efficient initiator to subsequently grow PS chains. In this case, the PS chains were grown preferentially from the R-groups present in R_2_Zn and also partially from the Me_3_SiCH_2_-moieties [42]. Thus, following completion of the CCTP reaction, the anionic initiator Me_3_SiCH_2_Li·(pmdeta) (150 μmol, [Li]/[Zn] = 1.0) and the styrene monomer (7.8 g, [styrene]/[Zn] = 500) were introduced sequentially, with 30 min between addition of the initiator and the monomer. The amount of Me_3_SiCH_2_Li·(pmdeta) was controlled not to be higher than the amount of Zn (i.e., [Li] ≤ [Zn]) to avoid formation of PS growing site of free styryl-Li. At the condition of [Li] ≤ [Zn], we presume that the PS chains were grown solely from the zincate species, which underwent alkyl exchange reaction with dialkylzinc species. Immediately following the introduction of Me_3_SiCH_2_Li·(pmdeta), the original colorless solution developed a deep yellow color due to generation of the styryl anion, which was caused by Me_3_SiCH_2_Li·(pmdeta) attacking the styrene moieties at the ends of the PO chains. However, this yellow color decreased in intensity with time, likely due to the formation of a zincate species through attack of the generated styryl anions at the Zn centers [50]. As a result, the viscosity of the product mixture also increased with time. Because the conversion of Me_3_SiCH_2_Li·(pmdeta) to styryl-Li·(pmdeta) was observed to some degree, we investigated whether styryl-Li·(pmdeta) could also trigger PS chain growth from R_2_Zn. Thus, when styrene polymerization was performed by the addition of a model compound of the styryl anion (i.e., CH_3_CH(Ph)-Li·(pmdeta), 0.50 equiv/Zn) to a methylcyclohexane solution containing styrene (500 equiv/Zn) and (hexyl)_2_Zn, the generation of PS with a narrow molecular weight distribution (*M*_w_/*M*_n_ = 1.25) was observed, where *M*_n_ = 21 kDa. The [PS growing sites]/[Zn] value calculated by the formula: [styrene]/[Zn]/DP (DP = *M*_n_/104), was 2.5, indicating that the PS chains were grown from all hexyl groups of (hexyl)_2_Zn, as well as from the initiator CH_3_CH(Ph)-Li·(pmdeta) (Scheme 4). This result indicates that the conversion of the fed Me_3_SiCH_2_Li·(pmdeta) to styryl-Li·(pmdeta) is not relevant in the growth of PS chains from (polymeryl)_2_Zn.

Upon the subsequent addition of the styrene monomer, the solution formed a deep yellow color, which persisted during the remainder of the polymerization process, thereby indicating that the active anionic styryl species was not destroyed even at a high polymerization temperature of 100–110 °C. The fed styrene was completely converted to PS, giving PS contents between 32 and 42 wt % based on the quantities of POs generated during the CCTP process. We expected that the excess styrene moieties present at the ends of PO chains participated in the polymerization of styrene as a comonomer, thereby resulting in attachment of PS chains at both ends of the PO chains.

In the ^1^H NMR spectra, both PS and PO signals were clearly observed at 7.2–6.5 ppm and 1.6–0.9 ppm, respectively (Figure S4). The selective extraction of PS homopolymers from the generated block copolymers was then attempted by a method previously developed in our group [42]. In the ^1^H NMR spectra of the extracted polymers (Figure S5), the Me_3_SiCH_2_-end group signals were clearly observed at ~0 ppm, thereby indicating that homo-PS was grown from the Me_3_SiCH_2_Li initiator. In addition, PO-related signals were observed at 1.2 and 0.9 ppm. Furthermore, in the gel permeation chromatography (GPC) traces, rather broad molecular weight distributions were observed (i.e., *M*_w_/*M*_n_ = 2–3) with distinct bimodal curves in many cases (entries 1, 3–4, 6–9, Table 1; Figure S6). The obtained NMR and GPC data therefore indicated that the block copolymers were extracted together with homo-PS. The *M*_n_ values of the homo-PS after elimination of the high molecular weight block copolymer by deconvolution ranged from 15 to 18 kDa (*M*_w_/*M*_n_ = 1.13–1.25), which corresponded with the values expected, assuming that PS chains were grown from all Zn-alkyl and Li sites (i.e., (PS weight, g)/(Li (mol) + 2 × Zn (mol)) = 7.8 g/(150 μmol + 2 × 150 μmol) = 17 kDa).

The homo-PS content (i.e., extracted PS (g)/total PS (g)) was particularly low (~23%). In this context, if Me_3_SiCH_2_Li·(pmdeta) is not involved in the reaction with the styrene moieties at the ends of the PO chains, the content of homo-PS grown from Me_3_SiCH_2_Li·(pmdeta) should be 33% (i.e., [Me_3_SiCH_2_Li·(pmdeta)]/([Me_3_SiCH_2_Li·(pmdeta)] + 2 × [Zn]) = 150 μmol/(150 μmol + 2 × 150 μmol)). The observed lower homo-PS contents therefore conform with the aforementioned conversion of portions of the fed Me_3_SiCH_2_Li·(pmdeta) to styryl-Li·(pmdeta) through attack on the styrene moieties at the ends of PO chains. In contrast, when a model compound of styryl anion (i.e., CH_3_CH(Ph)-Li·(pmdeta)) was employed as the initiator instead of Me_3_SiCH_2_Li·(pmdeta), the homo-PS content was high (33%; entry 11).

### 3.3. Characterization of the Block Copolymers

**Figure 2:**
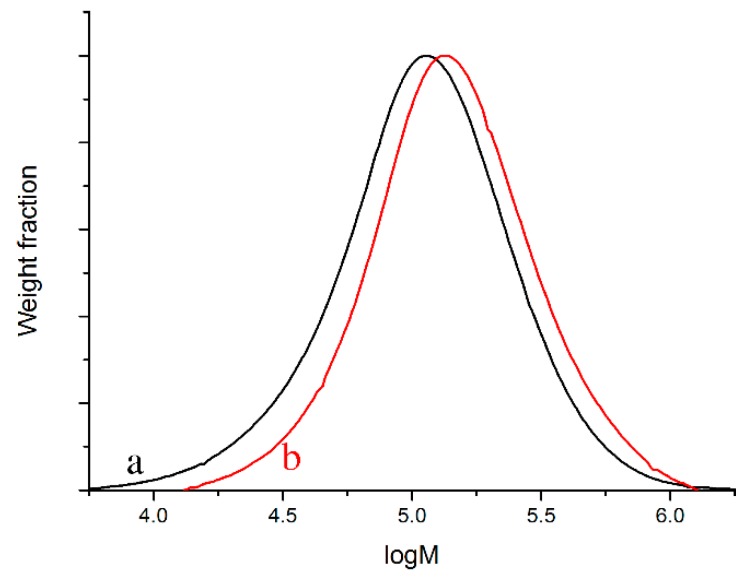
Gel permeation chromatograms (GPC) curves for the samples recorded before (**a**) and after (**b**) the anionic polymerization stages (entry 5, Table 1).

Response of the refractive index (RI) detector in the GPC instrument is opposite for the PO and PS samples (Figure S7), and so the quantitative analyses of the data are not reliable. However, as the attachment of PS-blocks should result in shorter retention time, the observed increases in both the *M*_n_ and *M*_w_ values after anionic styrene polymerization (Δ*M*_n_ = 27–54 kDa; Δ*M*_w_ = 29–61 kDa) confirmed their successful preparation. Upon shortening the time interval between the addition of Me_3_SiCH_2_Li·(pmdeta) and the styrene monomer to 10 min (entry 9), the *M*_n_ value was increased marginally (Δ*M*_n_ = 15 kDa; Δ*M*_w_ = 13 kDa). In addition, the GPC curves showed a clear overall shift after the anionic polymerization of styrene (Figure 2 and Figure S8), although the shift pattern differed somewhat from that observed in the batches prepared using the multinuclear Zn species Et-[Zn-(CH_2_)_6_]_3_-Zn-Et (**2**, Scheme 1c) [42]. In the latter case, regular-sized PS chains were attached at both ends of PO chains (hence, Δ*M*_n_ ≈ 2 × homo-PS *M*_n_), and the shift in the GPC curves plotted on the log(molecular weight) scale was substantial at low molecular weights, but was negligible upon moving to high molecular weights. However, in the cases where **3** was used, the shifts in the GPC curves plotted on the log(molecular weight) scale were substantial at both low molecular weights and high molecular weights (Figure 2), and the molecular weight distributions were also broader (*M*_w_/*M*_n_ = 1.67–1.84) than those of the batches obtained using Et-[Zn-(CH_2_)_6_]_3_-Zn-Et (*M*_w_/*M*_n_ = 1.37–1.64). These data suggest that the generated block copolymers are not simple PS-*block*-PO-*block*-PS triblocks but instead contain higher blocks (e.g., PS-*block*-PO-*block*-PS-*block*-PO-*block*-PS), which can be generated when the chain-growing styryl anion connected to the PO chains attacks the styrene moieties at the ends of other PO chains. In an attempt to prepare diblock copolymer using Et_2_Zn instead of **3**, the molecular weight increment was marginal (Δ*M*_w_ = 1 kDa) because of aforementioned malfunction in the CCTP process.

**Figure 3:**
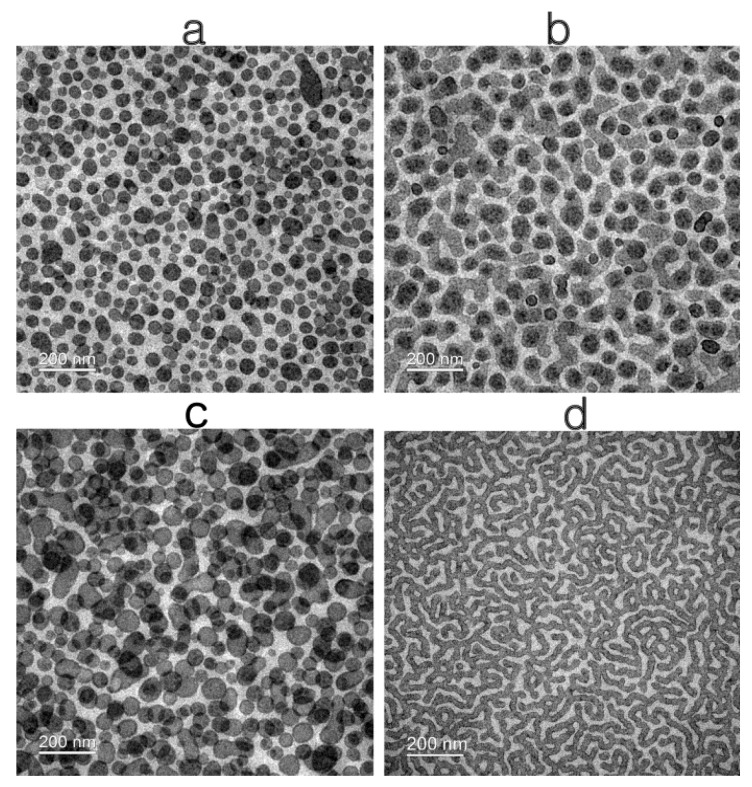
Transmission electron microscopy (TEM) images of the prepared block copolymers: (**a**) entry 1, (**b**) entry 3, (**c**) entry 5 (Table 1), and (**d**) commercial-grade PS-*block*-poly(ethylene-*co*-1-butene)-*block*-PS (SEBS).

Phase separation between the PS and PO fragments was clearly visible by the transmission electron microscopy (TEM) after selectively staining the PS domains with RuO_4_ vapor (Figure 3). More specifically, spherical PS domains measuring several tens of nm in size were distributed in the PO matrix in the sample containing a low propylene fraction (*F*_C3_ = 0.22; entry 1; Figure 3a). Upon increasing the propylene fraction of the PO matrix, a number of these spherical domains collapsed to form wormlike structures (Figure 3b,c). However, this phase separation was not as regular as that exhibited by commercial-grade SEBS (Figure 3d). In addition, the prepared triblock copolymers did not exhibit the distinct glass transition (*T*_g_) signal of PS at ~100 °C in the differential scanning calorimetry (DSC) thermogram (Figure S9) due to the observed phase separation. Weak and broad melting signals were observed in the range of 0–90 °C, as also observed in the DSC thermogram of SEBS.

**Figure 4:**
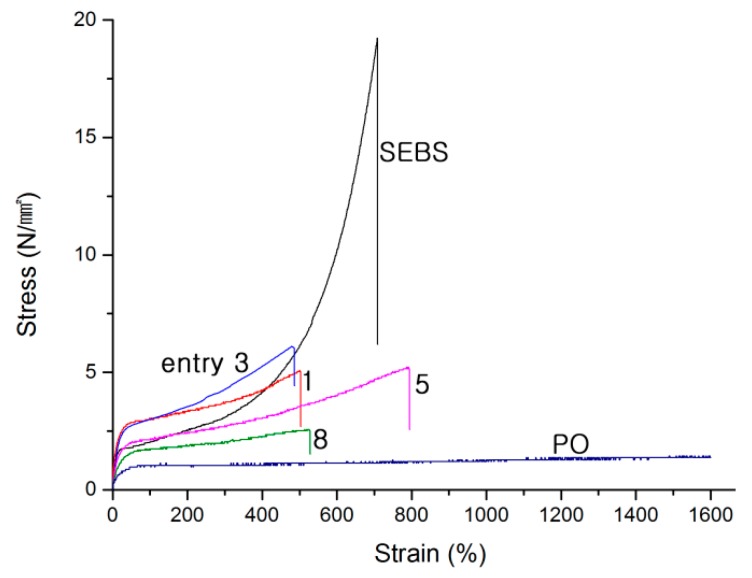
Tensile stress-strain curves for the prepared block copolymers (entries 1, 3, 5, and 8, Table 2), poly(ethylene-*co*-propylene), and SEBS.

**Figure 5:**
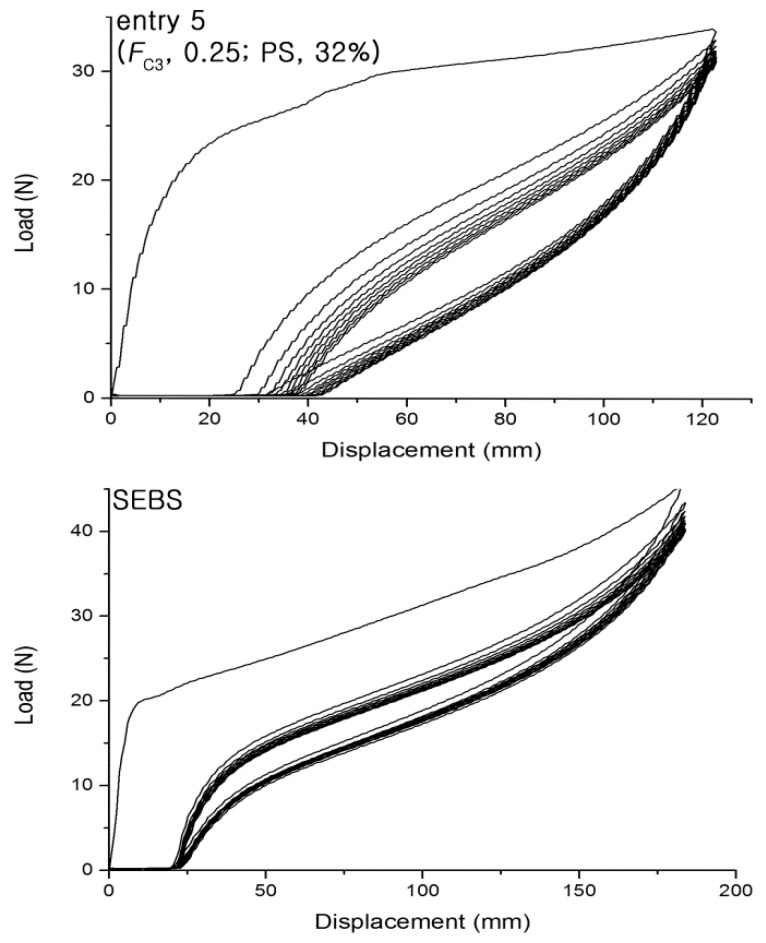
Plots of the cyclic tensile tests.

We then measured tensile properties of the prepared block copolymers following compression of the polymer lumps into films of ~1 mm thickness (Table 2 and Figure 4). As indicated, the tensile strengths were weak (2–3 N/mm^2^) at high propylene fractions (*F*_C3_ = 0.28–0.30) and the elongation at breaking point percentage values were low at high PS contents (e.g., 490% versus 770% at 38% and 32% PS contents, respectively; entry 4 versus 5). An outlier was observed for one sample (entry 2), where a low tensile strength (2.68 N/mm^2^) and low elongation at breaking point (300%) were observed even for a low propylene fraction (*F*_C3_ = 0.22) and a low PS content (33%). The GPC data of that sample also deviated from the normal, where Δ*M*_w_ (61 kDa) was significantly higher than Δ*M*_n_ (31 kDa). Such differences are likely due to greater quantity of higher blocks (e.g., PS-*block*-PO-*block*-PS-*block*-PO-*block*-PS) present in this sample. For the remainder of the samples, the values of Δ*M*_w_ and Δ*M*_n_ did not differ significantly. In addition, for the samples exhibiting a low Δ*M*_n_ (entry 9) and a high amount of homo-PS (entry 11), the tensile strength was particularly low (<2.0 N/mm^2^). The optimal data were recorded for the sample with an *F*_C3_ value of 0.25 and a styrene content of 33% (entry 5) as this sample exhibited a high elongation at breaking point (770%) comparable to that of commercial-grade SEBS (720%), although its tensile strength was lower. Indeed, SEBS is also known to exhibit an optimal elastomeric performance at *F*_C3_ = 0.25 and with a styrene content of 33%. In the cyclic tensile tests, the prepared triblock copolymers exhibited elastomeric behavior with no break over 10 cycles, which is comparable to the behavior of commercial-grade SEBS (Figure 5 and Figure S10). However, the sample containing a high quantity of homo-PS (33%) was broken prior to reaching 10 cycles (entry 11). For all other samples, in the first cycle, a degree of permanent deformation was observed, although this was followed by minimal deformation in subsequent cycles. This permanent deformation was somewhat greater in the prepared block copolymers than in commercial-grade SEBS.

## 4. Conclusions

Dialkylzinc containing styrene moieties ((CH_2_=CHC_6_H_4_CH_2_CH_2_)_2_Zn) was prepared by an alkyl exchange reaction between the hydroboration product of divinylbenzene (CH_2_=CHC_6_H_4_CH_2_CH_2_-BEt_2_) and Et_2_Zn. Poly(ethylene-*co*-propylene) chains were then grown from (CH_2_=CHC_6_H_4_CH_2_CH_2_)_2_Zn via CCTP using a pyridylaminohafnium catalyst to afford di-end functional PO chains functionalized with styrene and Zn moieties. The measured molecular weights (*M*_n_) of the POs generated via these CCPT reactions corresponded with the expected values calculated using the following formula: PO weight (g)/(2 × Zn (mol)). PS chains were subsequently grown from the generated di-end functional PO chains by introduction of styrene monomers in addition to the anionic initiator Me_3_SiCH_2_Li·(pmdeta). Counting of the PS growth sites from the measured *M*_n_ of the extracted homo-PS (7.8 g/17 kDa = 450 μmol) indicated that the PS chains were grown from all Zn-alkyl sites and Li sites (2 × 150 μmol-Zn + 150 μmol-Li). In addition, the obtained homo-PS content was low (~23%) due to the conversion of portions of the fed Me_3_SiCH_2_Li·(pmdeta) into styryl-Li·(pmdeta) species by attack at the styrene moieties at the end of the PO chains. The molecular weights of the obtained block copolymers increased after styrene polymerization (Δ*M*_n_ = 27–54 kDa). TEM images showed spherical and wormlike PS domains measuring several tens of nm, which were segregated in the PO matrix. In the cyclic tensile test, the majority of prepared copolymers exhibited thermoplastic elastomeric properties with no break being observed over 10 cycles, which is comparable performance to commercial-grade SEBS. The optimal tensile properties were observed for the sample containing a propylene fraction of 0.25 and a styrene content of 33% (tensile strength = 5.2 N/mm^2^; elongation at breaking point = 770%).

## Figures and Tables

**Table 1:** Results of the sequential CCTP and anionic styrene polymerization process. ^a^

Entry	Yield (g)	*F*_C3_^b^	(PS, g)/(Total, g)	(Homo-PS, g)/(PS, g)	Homo-PS *M*_n_ (kDa); PDI ^c^	PO *M*_n_ (kDa); PDI ^d^			Block Copolymer *M*_n_ (kDa); PDI ^d^
						Expected ^e^	PO Equiv ^f^	PS Equiv ^d^	
1	21.3	0.22	0.37	0.17	16; 1.20	45	52; 1.92	90; 1.95	123; 1.73
2	23.9	0.22	0.33	0.24	23; 2.01	54	48; 1.88	84; 1.90	120; 1.84
3	18.7	0.24	0.42	0.17	15; 1.13	36	47; 1.89	84; 1.92	111; 1.71
4	20.1	0.27	0.38	0.20	17; 1.17	41	54; 1.92	88; 1.95	125; 1.72
5	24.1	0.25	0.32	0.23	18; 1.46	54	47; 1.87	79; 1.88	109; 1.69
6	20.0	0.30	0.39	0.22	17; 1.15	41	51; 1.98	80; 2.00	134; 1.67
7	22.9	0.29	0.34	0.18	17; 1.25	50	50; 1.89	79; 1.91	106; 1.76
8	24.7	0.28	0.32	0.19	18; 1.25	56	50; 1.96	80; 2.00	110; 1.75
9	22.8	0.30	0.34	0.18	16; 1.17	50	49; 1.88	79; 1.91	94; 1.74
10	21.9	0.28	0.36	0.24	19; 1.55	47	50 ;1.87	80; 1.90	105; 1.79
11 ^g^	23.8	0.29	0.33	0.33	25; 1.68	53	51; 1.81	82; 1.83	114; 1.63

^a^ Polymerization conditions: **3** (150 μmol), catalyst **4** (4.0 μmol) activated with [(C_18_H_37_)_2_MeNH]^+^[B(C_6_F_5_)_4_]^−^ (1.0 equiv), 95–110 °C, 40 min, then sequential addition of Me_3_SiCH_2_Li·(pmdeta) (150 μmol) and styrene (7.8 g, 500 equiv per Zn) with a time difference of 30 min (10 min for entry 9, 60 min for entry 10), 100–110 °C, 5.0 h (for complete conversion of styrene). ^b^ The propylene mole fraction in POs ([C3]/([C2] + [C3]) was measured by ^1^H NMR spectroscopy. ^c^ Measured by GPC at 40 °C eluting with toluene and using PS-standards. ^d^ Measured by GPC at 160 °C eluting with 1,2,4-trichlorobenzene and using PS standards. ^e^ (PO, g)/(2 × Zn (mol)). ^f^ Converted to PO equivalents by universal calibration. ^g^ CH_3_CH(Ph)Li·(pmdeta) was used instead of Me_3_SiCH_2_Li·(pmdeta).

**Table 2:** Results of the tensile test and the cyclic tensile test.

Entry	*F*_C3_	PS (%)	*M*_n_ (kDa); PDI	Tensile Test		Cyclic Tensile Test	
				Tensile Strength (N/mm^2^)	Elongation at Break (%)	Elastic Recovery at 1st Cycle (%)	Elastic Recovery at 10th Cycle (%)
1	0.22	37	121; 1.73	5.09	490	65	55
2	0.22	33	120; 1.84	2.68	300	90	85
3	0.24	42	111; 1.71	6.10	470	61	54
4	0.27	38	125; 1.72	4.25	490	78	66
5	0.25	32	109; 1.69	5.22	770	77	64
6	0.30	39	134 1.67	2.55	290	88	84
7	0.29	34	106; 1.76	2.90	480	91	81
8	0.28	32	110; 1.75	2.56	520	85	74
9	0.30	34	94; 1.74	1.82	440	81	75
10	0.28	36	105; 1.79	2.69	550	85	76
11	0.29	33	114; 1.63	1.64	220	81	broken
12	SEBS	-	-	19.2	720	89	86

## Supplementary Materials

The following are available online at http://www.mdpi.com/2073-4360/9/10/481/s1, Figure S1: ^1^H NMR spectra of poly(ethylene-*co*-propylene) generated in the presence of styrene (**a**) and in the absence of styrene (**b**), Figure S2: ^1^H NMR spectra of a low molecular weight poly(ethylene-*co*-propylene), which was synthesized by feeding a high amount of **3** (Zn = 500 μmol) and by cutting the polymerization at an early stage (5 min), Figure S3: ^1^^3^C NMR spectrum of **3** in C_6_D_6_, Figure S4: ^1^H NMR spectrum of block copolymer in C_6_D_6_ at 70 °C (entry 5 in Table 1), Figure S5: ^1^H NMR spectrum of the extracted PS homopolymer, Figure S6: GPC curve of the extracted PS homopolymer (entry 1 in Table 1), Figure S7: GPC curves for PO and PS samples showing that the RI detector response is opposite, Figure S8: GPC curves recorded before and after the anion styrene polymerization, Figure S9: DSC thermograms for triblock copolymers, Figure S10: Plots of the cyclic tensile test for triblock copolymers.
