# Supplementary material for: Polystyrene Chain Growth from Di-End-Functional Polyolefins for Polystyrene-Polyolefin-Polystyrene Block Copolymers
Source: Polymers (Basel). 2017 Oct 2;9(10):481. doi: 10.3390/polym9100481 (PMC6418507; doi:10.3390/polym9100481)
Supplement: Supplementary file 1 [file polymers-09-00481-s001.pdf]

Supplementary Materials: Polystyrene Chain Growth from Di-end-functional Polyolefins for Polystyrene-Polyolefin-Polystyrene Block Copolymers

Chung Sol Kim, Seung Soo Park, Sung Dong Kim, Su Jin Kwon, Jun Won Baek and Bun Yeoul Lee

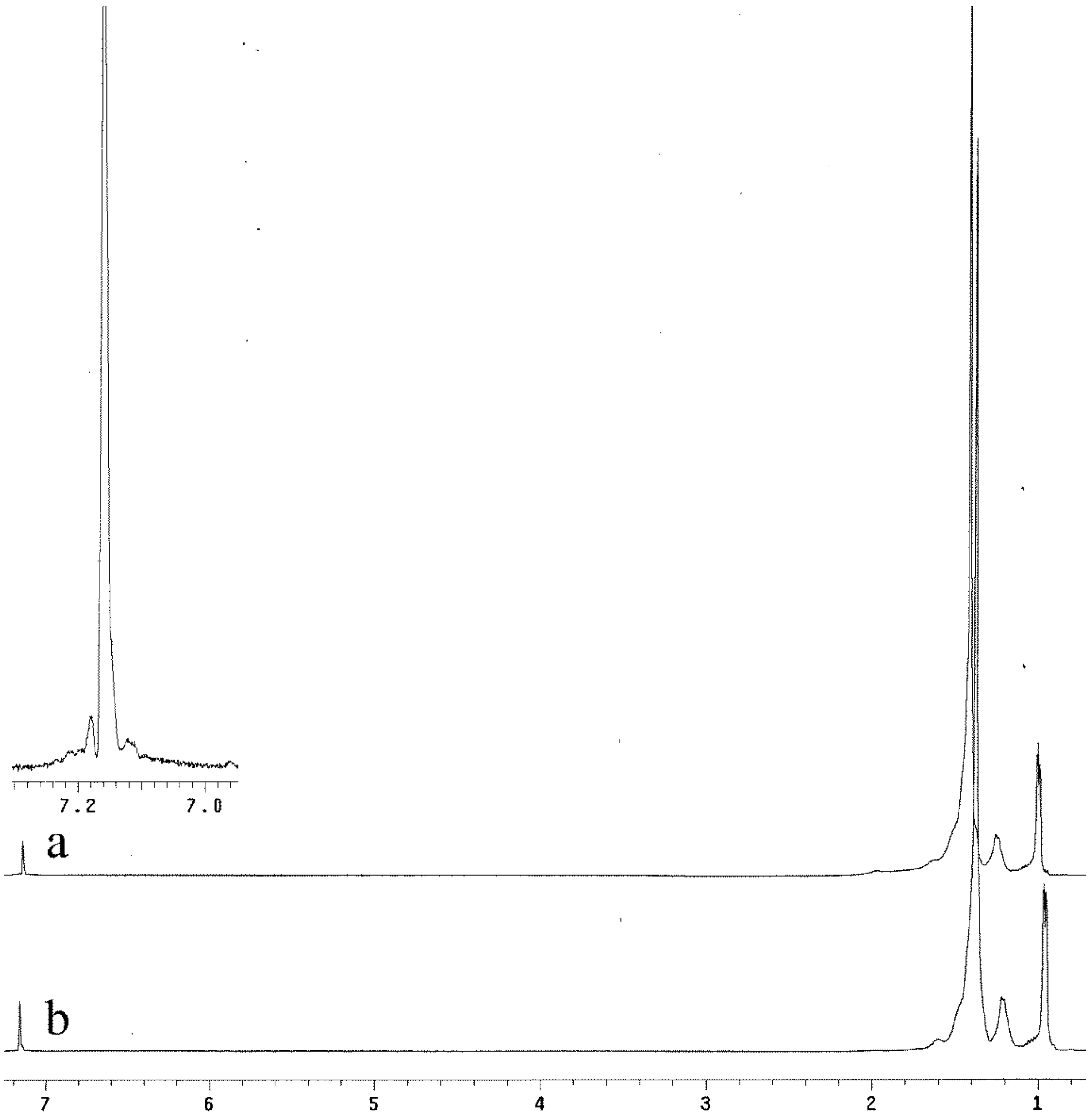

Figure S1. ^{1}H NMR spectra of poly(ethylene-*co*-propylene) generated in the presence of styrene (**a**) and in the absence of styrene (**b**).

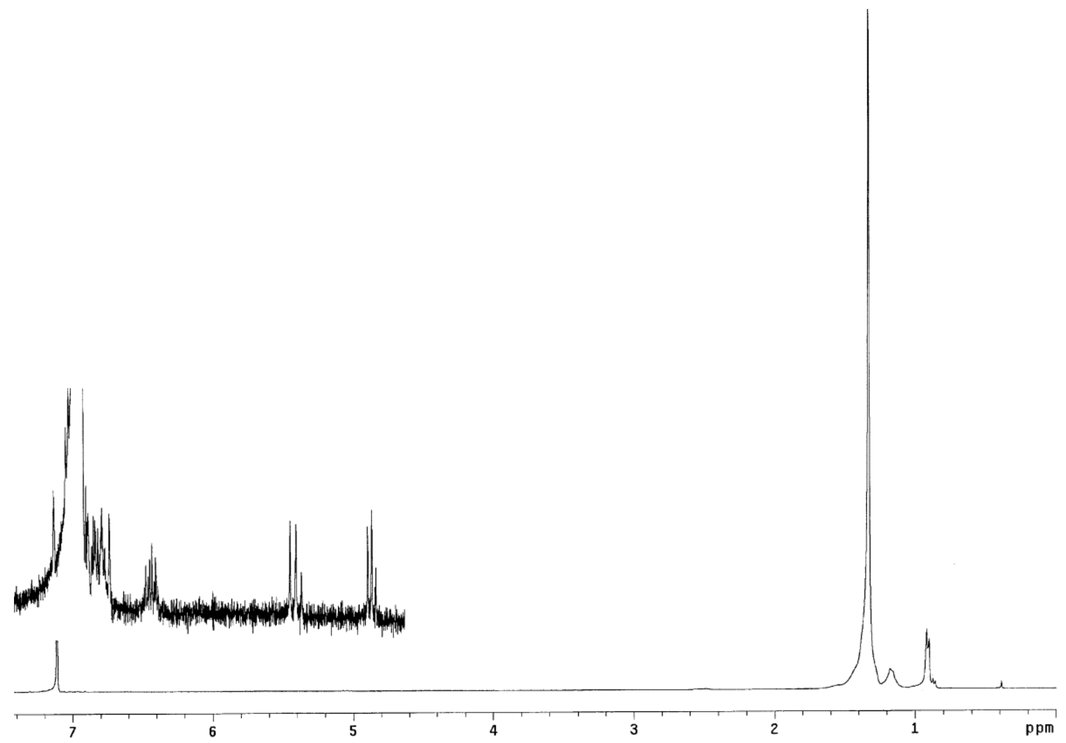

Figure S2. ^{1}H NMR spectra of a low molecular weight poly(ethylene-*co*-propylene), which was synthesized by feeding a high amount of **3** (Zn = 500 μmol) and by cutting the polymerization at an early stage (5 min).

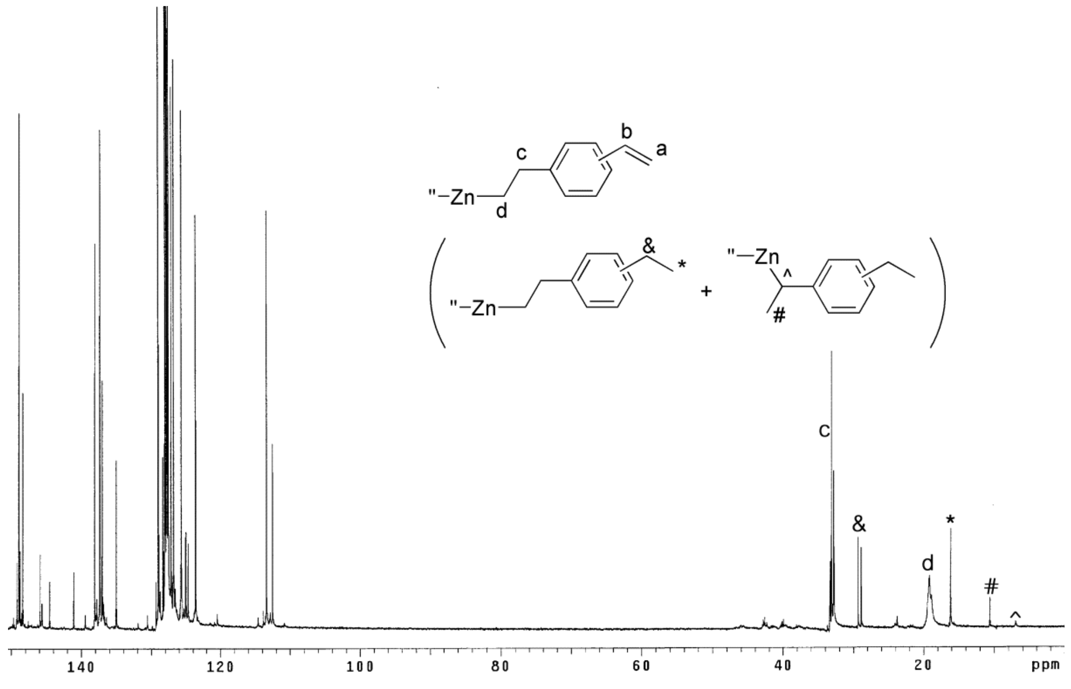

Figure S3. ^{13}C NMR spectrum of **3** in C_6D_6.

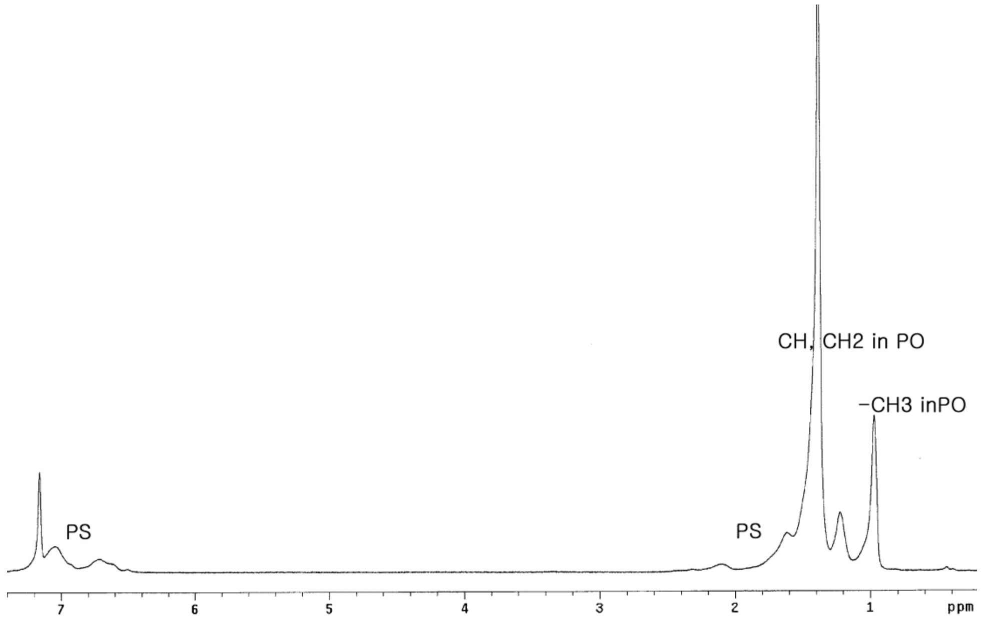

Figure S4. ¹H NMR spectrum of block copolymer in C_6D_6 at 70 °C (entry 5 in Table 1).

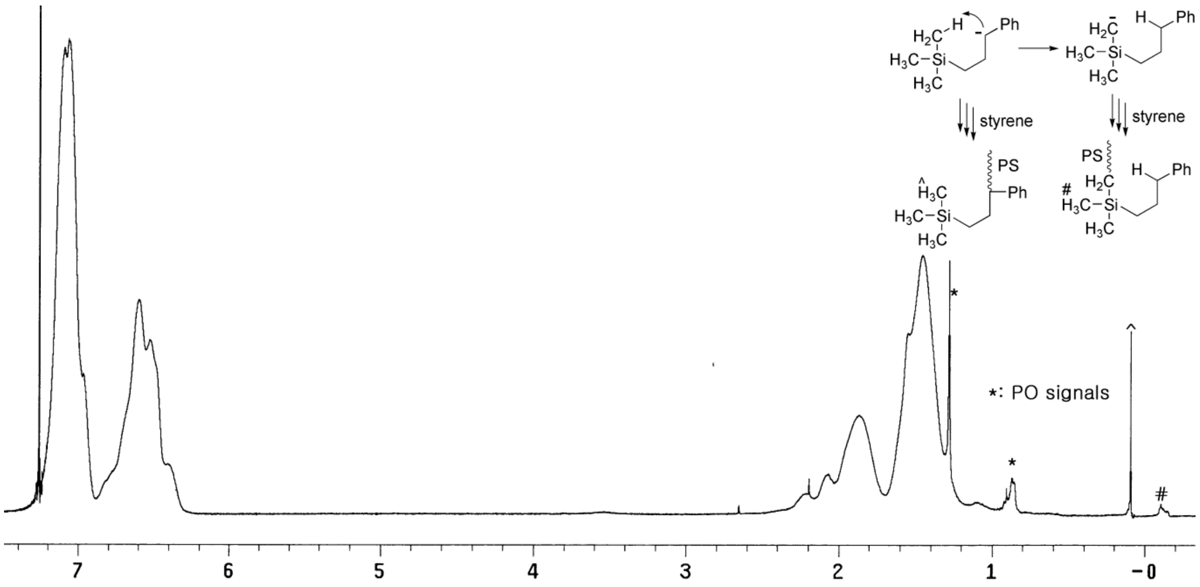

Figure S5. ¹H NMR spectrum of the extracted PS homopolymer.

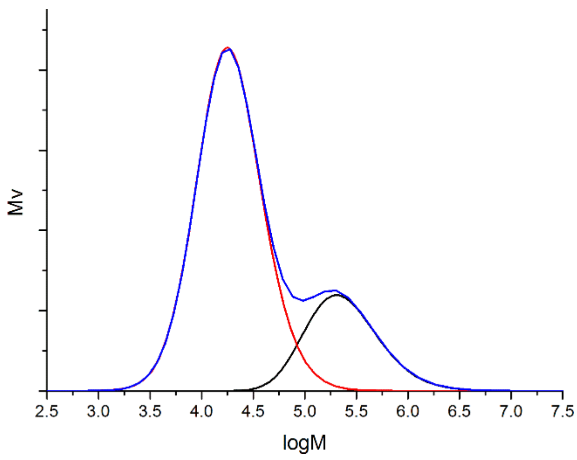

Figure S6. GPC Curve of the extracted PS homopolymer (entry 1 in table 1).

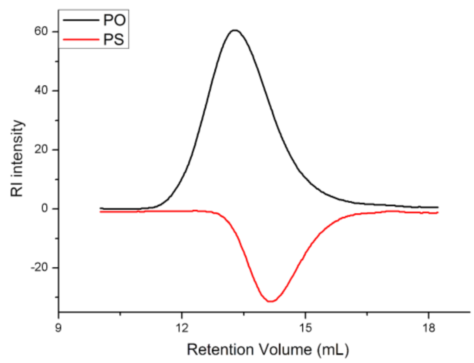

Figure S7. GPC curves for PO and PS samples showing that the RI detector response is opposite (weight concentration is the same for each).

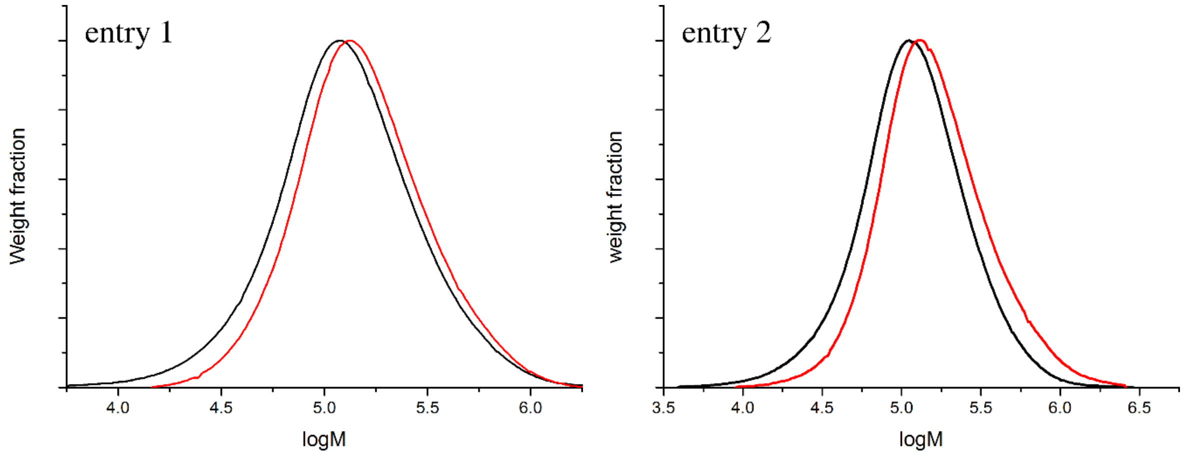

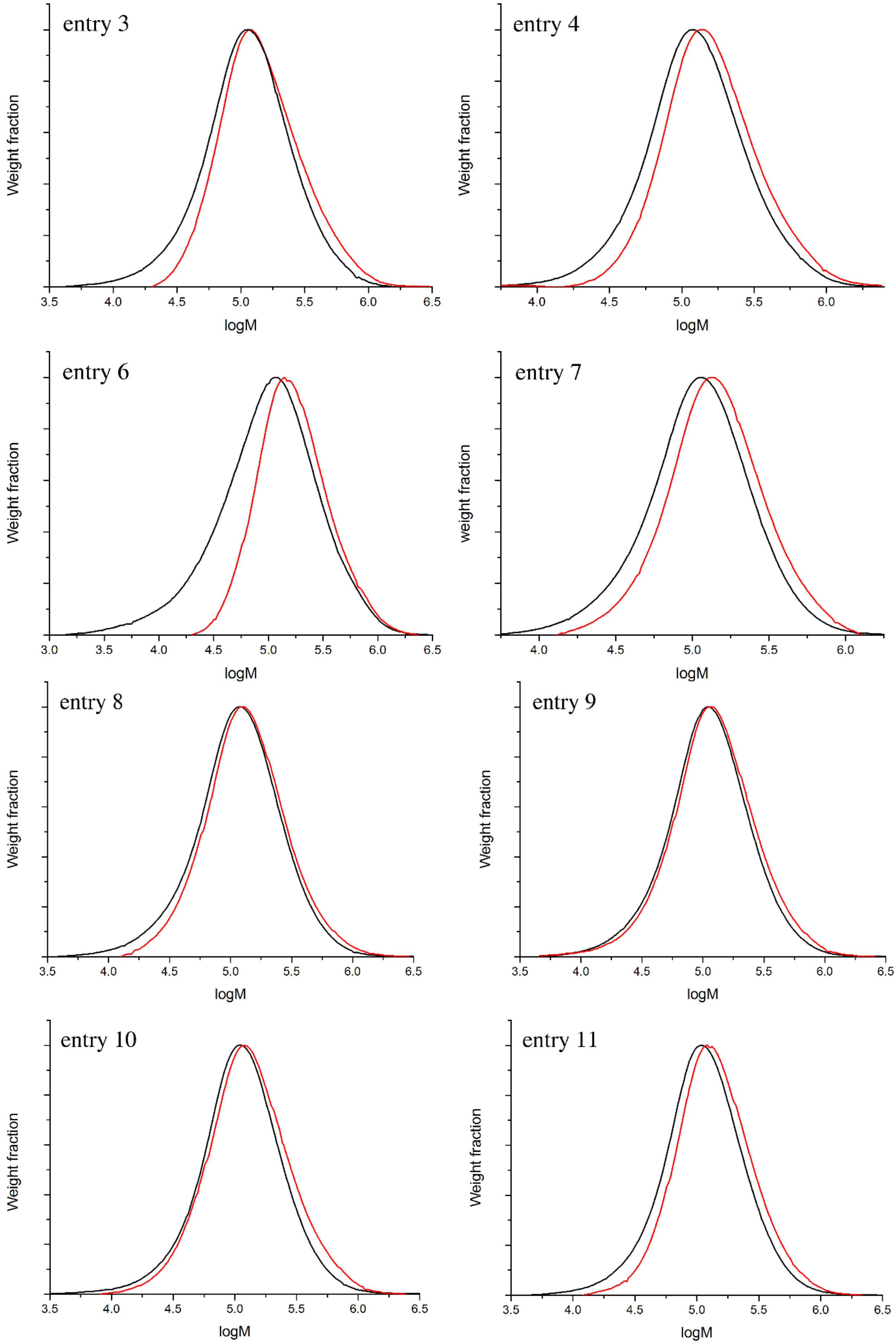

Figure S8. GPC curves recorded before and after the anion styrene polymerization.

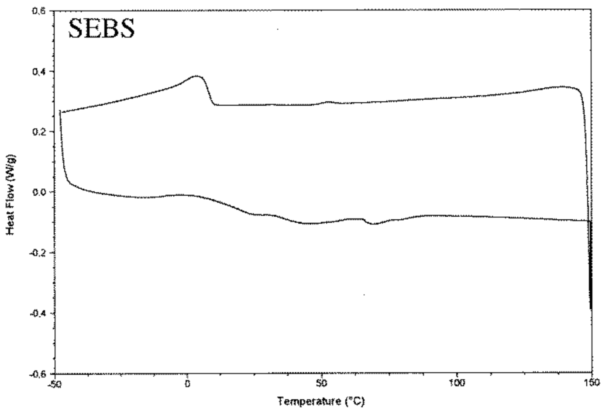

Poly(ethylene-*co*-propylene)

triblock copolymer

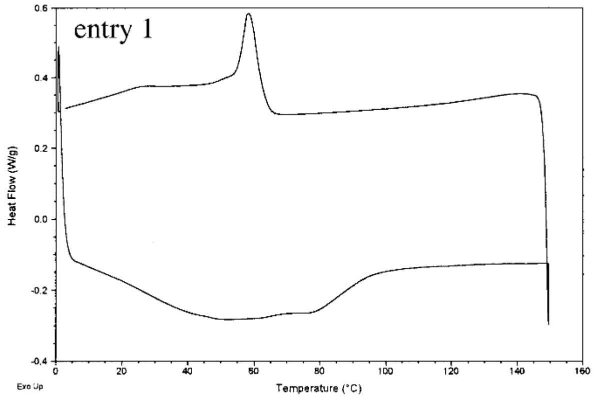

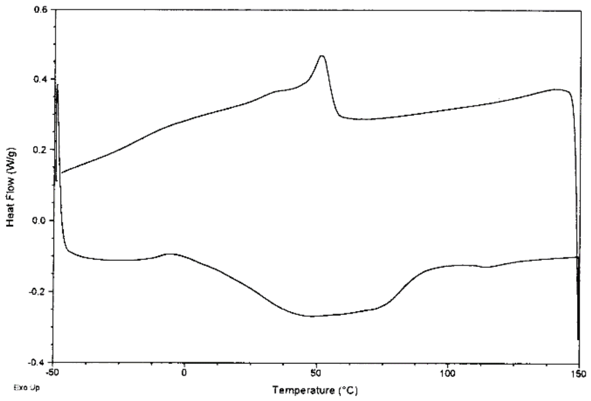

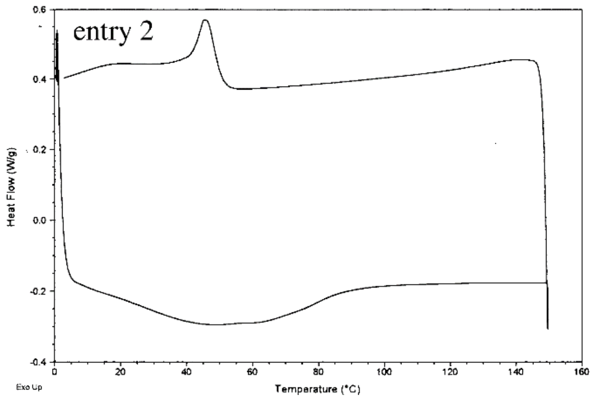

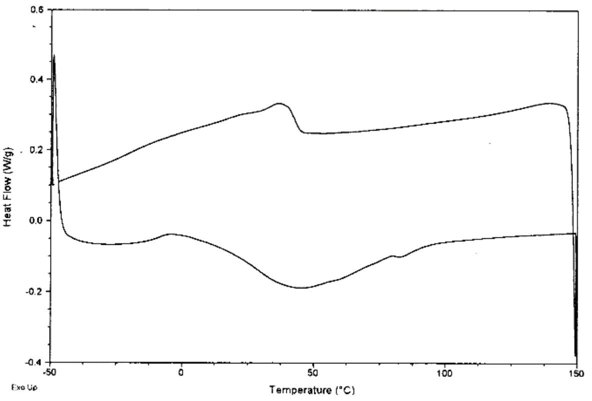

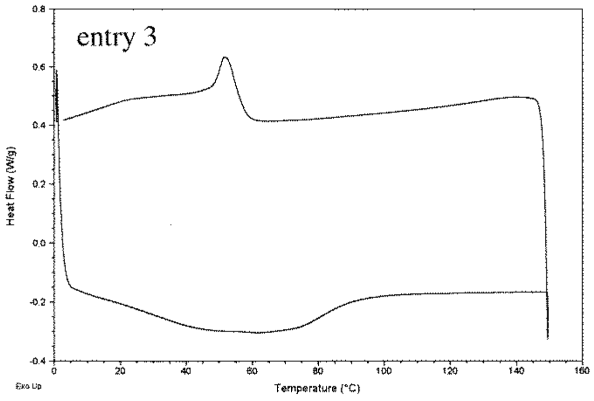
entry 3
Heat Flow (W/g)
Temperature (°C)
Exo Up

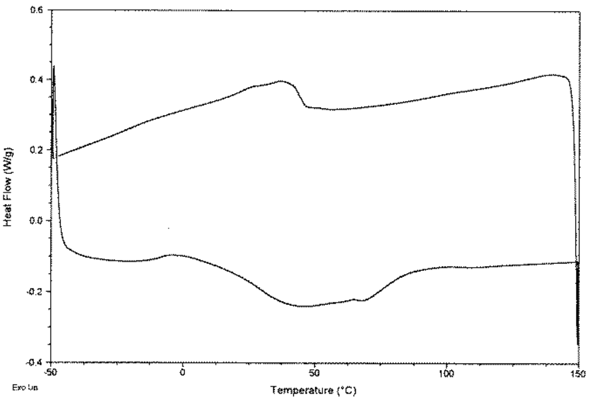
Heat Flow (W/g)
Temperature (°C)
Exo Up

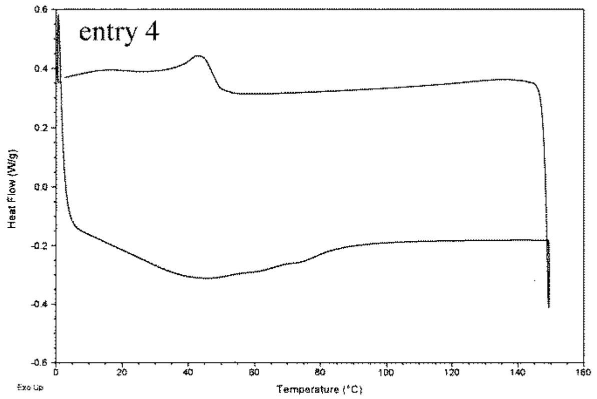
entry 4
Heat Flow (W/g)
Temperature (°C)
Exo Up

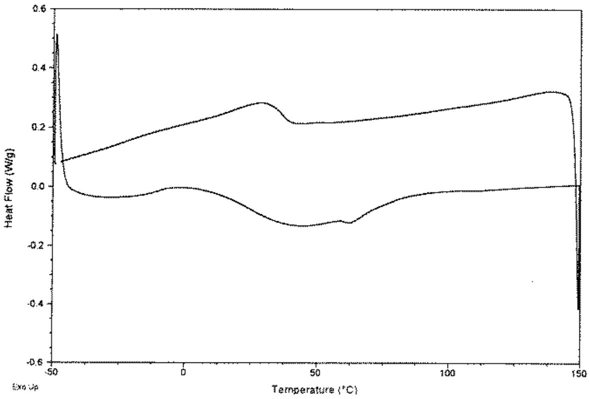
Heat Flow (W/g)
Temperature (°C)
Exo Up

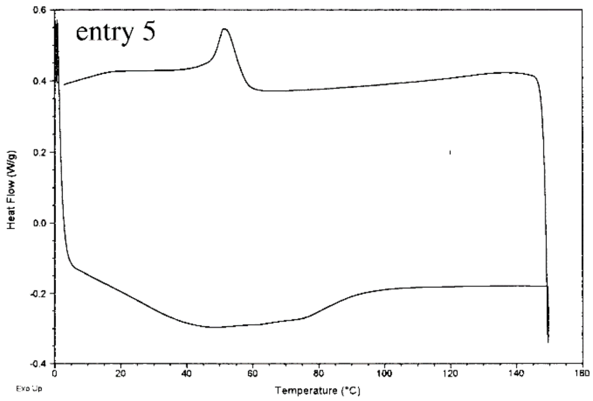
entry 5
Heat Flow (W/g)
Temperature (°C)
Exo Up

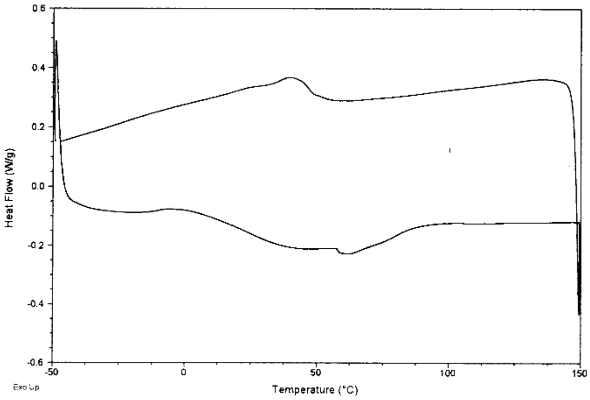
Heat Flow (W/g)
Temperature (°C)
Exo Up

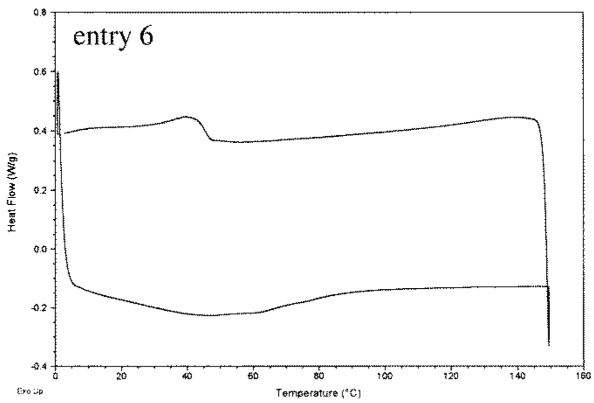
entry 6
Heat Flow (W/g)
Temperature (°C)
Exo Up

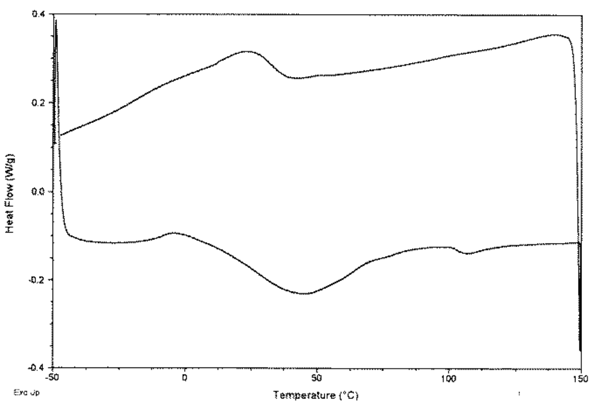
Heat Flow (W/g)
Temperature (°C)
Exo Up

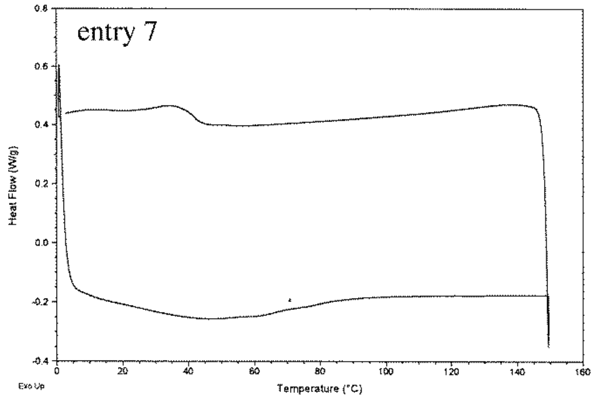
entry 7
Heat Flow (W/g)
Temperature (°C)
Exo Up

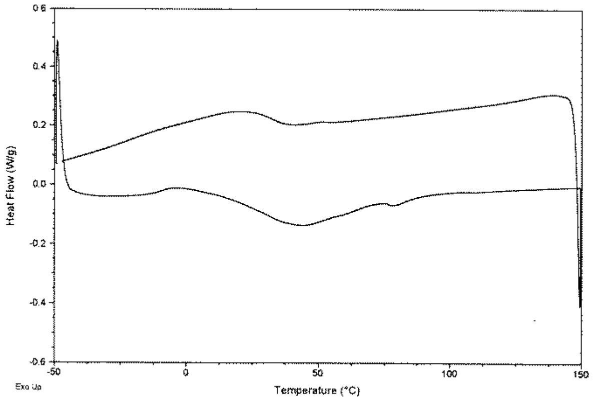
Heat Flow (W/g)
Temperature (°C)
Exo Up

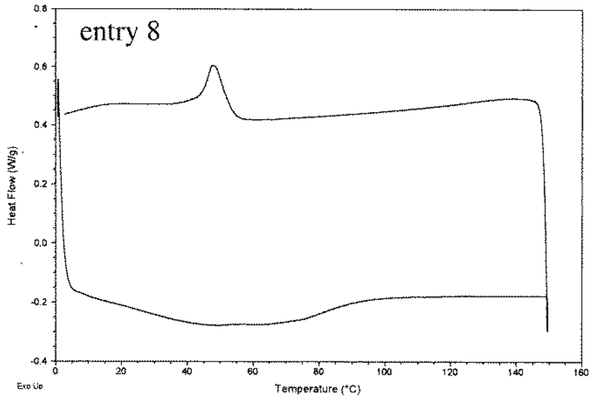
entry 8
Heat Flow (W/g)
Temperature (°C)
Exo Up

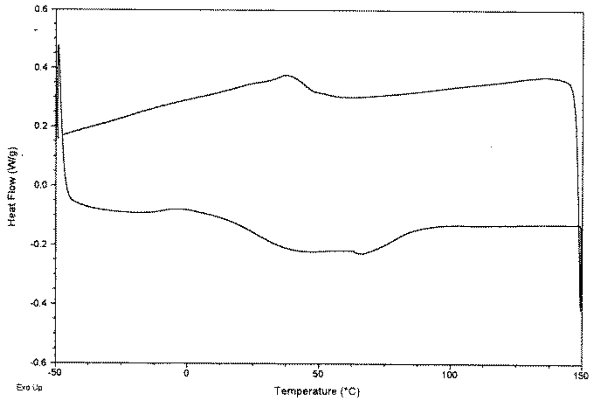
Heat Flow (W/g)
Temperature (°C)
Exo Up

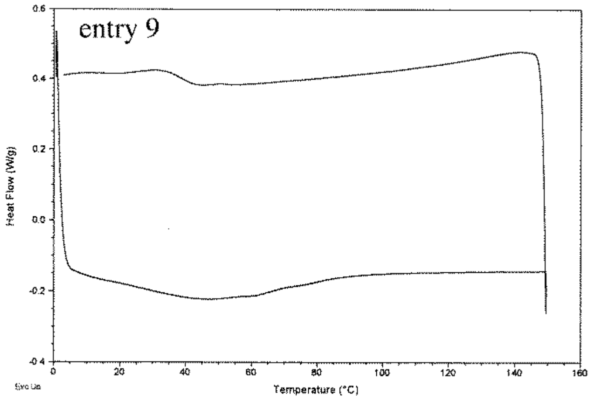
entry 9
Heat Flow (W/g)
Temperature (°C)
Exo Up

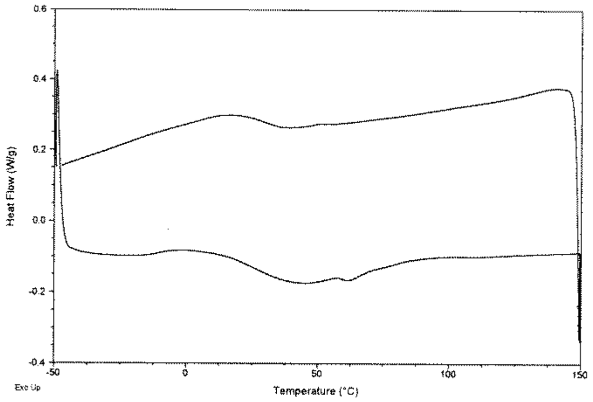
Heat Flow (W/g)
Temperature (°C)
Exo Up

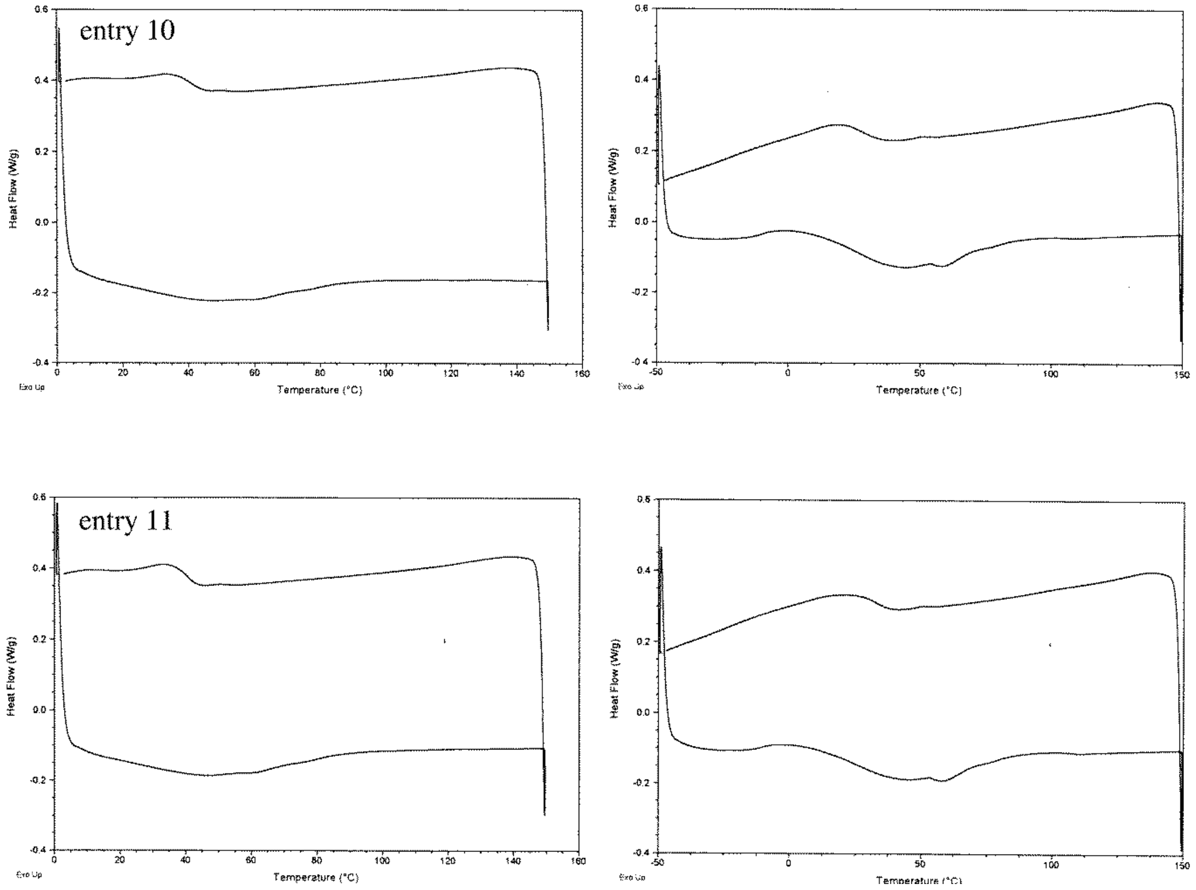

Figure S9. DSC thermograms for SEBS, poly(ethylene-*co*-propylene), and triblock copolymer.

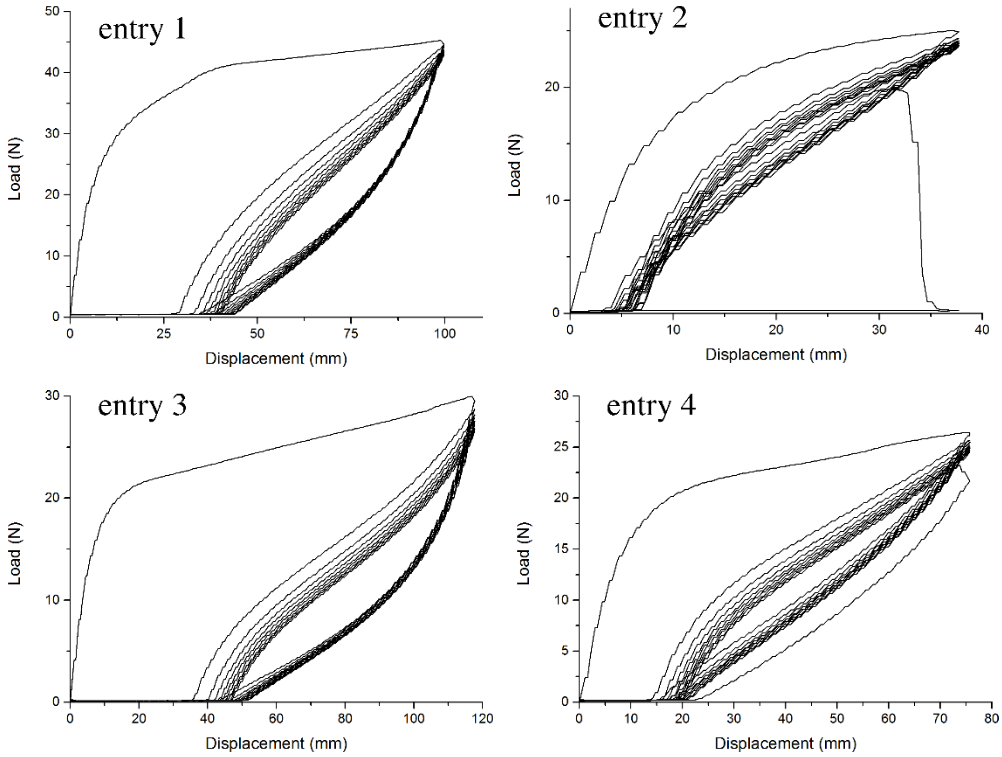

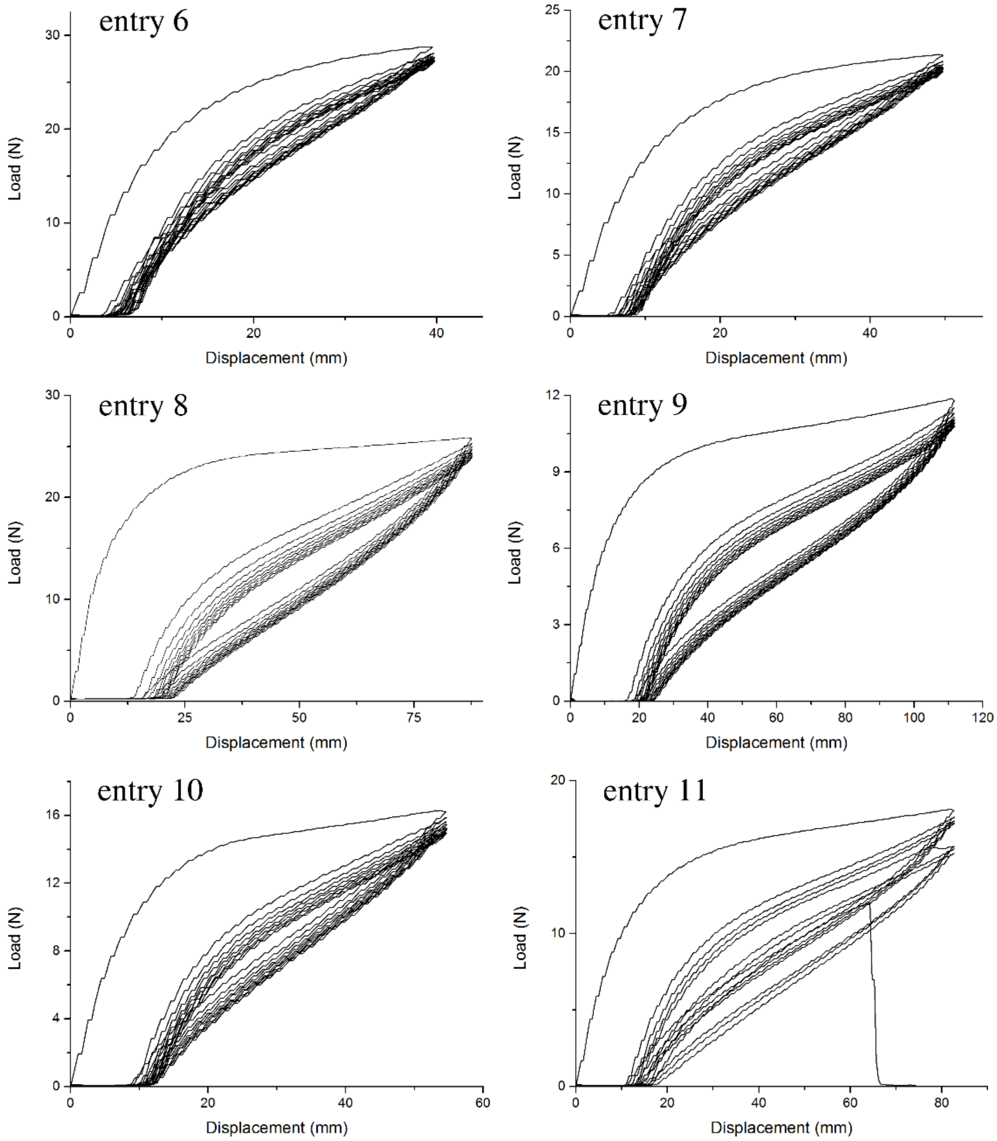

Figure S10. Plots of the cyclic tensile test for triblock copolymer.
